# k-t SENSE accelerated stress myocardial perfusion MRI at 3 Tesla

**DOI:** 10.1186/1532-429X-11-S1-O35

**Published:** 2009-01-28

**Authors:** Shingo Kato, Hajime Sakuma, Motonori Nagata, Nanaka Ishida, Kakuya Kitagawa, Masaki Ishida, Hiroshi Nakajima, Katsuya Onishi, Masaaki Ito, Kan Takeda

**Affiliations:** Mie University Hosiptal, Tsu, Mie Japan

**Keywords:** High Spatial Resolution, Heart Beat, Significant Coronary Artery Disease, Suspected Coronary Artery Disease, Image Quality Score

## Objective

The purpose of this study was to evaluate the feasibility and diagnostic accuracy of high spatial resolution stress myocardial perfusion MRI acquired at every heartbeat by using k-t SENSE and 3 Tesla MR imager.

## Background

High spatial and temporal resolutions are required for the accurate assessment of myocardial ischemia using stress perfusion MRI.

## Methods

Thirty-three patients with suspected coronary artery disease were studied. High spatial resolution (<2 mm) first-pass contrast enhanced MR images were obtained at rest and during stress by using a 3.0 T MR imager (Achieva) and k-t SENSE acceleration factor of 5. Saturation recovery TFE images were acquired with TR/TE of 2.9 ms/1.5 ms, FOV = 40 × 30 cm, matrix = 256 × 192, slice thickness = 8 mm. Three short-axis sections of the left ventricle were imaged at every heart beat. Two observers determined the image quality score (1:poor – 4:excellent) and recorded the presence or absence of respiratory artifacts and endocardial dark rim artifacts using a 16-segment model.

## Results

**Figure 1:**
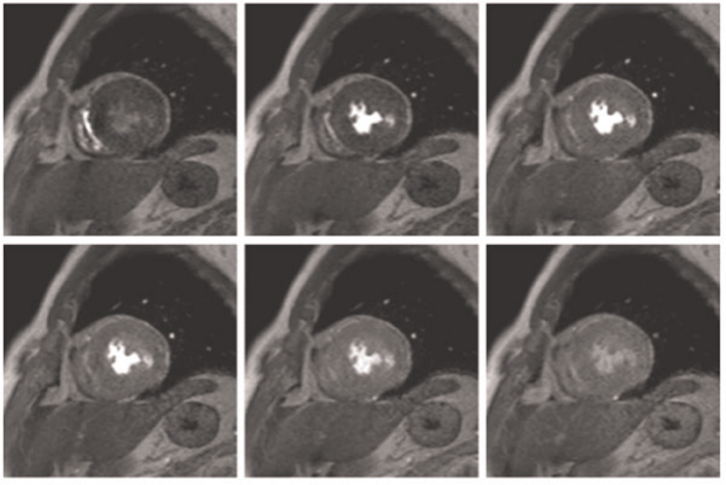
**Rest perfusion MRI acquired with 3 T MR imager, 32 channel cardiac coils and k-t SENSE in a patient with triple vessel disease**. Rest perfusion MRI is normal and no endocardial banding artifact is observed.

**Figure 2:**
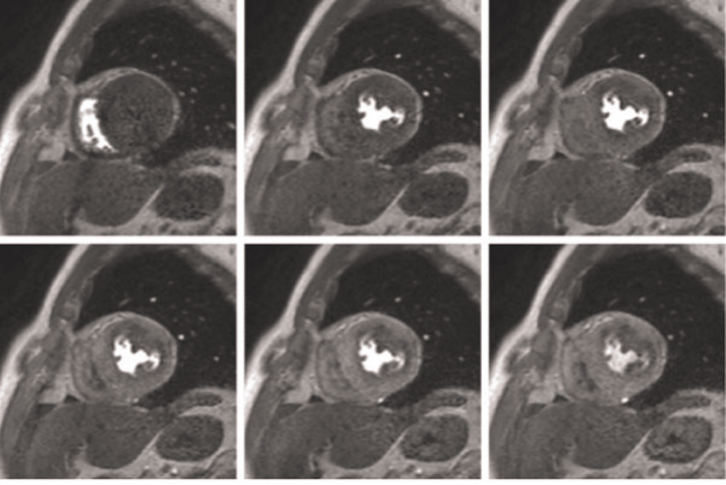
**Stress perfusion MRI in the same patient with triple vessel disease**. Subendocardial ischemia is clearly demonstrated in the anteroseptal well, lateral wall and inferior wall on high resolution images.

All studies were successfully completed, with the averaged image quality score of 3.8 ± 0.4. Endocardial dark rim artifacts were observed in 17 (3.2%) of 528 segments, but there were no cases in which dark-rim artifacts influenced the diagnosis. Respiratory artifacts were found in 11 (2.1%) of 528 segments. In 14 patients who underwent coronary angiography within 2 weeks from MR study, stress-rest perfusion MRI demonstrated the sensitivity, specificity, positive and negative predictive values and accuracy of were 90.9%(10/11), 96.7%(30/31), 90.9%(10/11), 96.7%(30/31) and 95.2%(40/42) for detecting significant coronary artery disease. Figures 1 and 2.

## Conclusion

Perfusion MR images with high spatial resolution can be acquired at every heart beat by using a 3 T MR imager and k-t SENSE acceleration. This approach can substantially reduce endocardial dark rim artifacts and allows for an accurate detection of myocardial ischemia in patients with flow-limiting coronary artery disease.

